# The Impact of Vitamin Deficiencies on Oral Manifestations in Children

**DOI:** 10.3390/dj12040109

**Published:** 2024-04-17

**Authors:** Stjepanka Lešić, Zrinka Ivanišević, Bruno Špiljak, Matej Tomas, Magdalena Šoštarić, Aleksandar Včev

**Affiliations:** 1 Department of Dental Medicine, Faculty of Dental Medicine and Health Osijek, J. J. Strossmayer University of Osijek, 31000 Osijek, Croatia; slesic@fdmz.hr (S.L.); zivanisevic@fdmz.hr (Z.I.); 2 School of Dental Medicine, University of Zagreb, 10000 Zagreb, Croatia; 3 Faculty of Dental Medicine and Health Osijek, J. J. Strossmayer University of Osijek, 31000 Osijek, Croatia; sostaricmagdalena@gmail.com; 4 Department of Pathophysiology, Physiology and Immunology, Faculty of Dental Medicine and Health Osijek, J. J. Strossmayer University of Osijek, 31000 Osijek, Croatia; avcev@fdmz.hr

**Keywords:** children, hypoplastic teeth, oral manifestations, vitamin deficiencies, dental health, nutritional education

## Abstract

Vitamins play a vital role in human health, particularly in the development and maintenance of oral health in children. These nutrients are broadly categorized into fat-soluble and water-soluble types, crucial for children’s well-being. The objective of this study is to investigate the impact of vitamin deficiencies on the oral health of children, focusing on how these deficiencies contribute to various oral health issues and determining the relationship between specific vitamin shortages and oral diseases. Findings indicate that shortages in vitamins A and D lead to enamel issues and a higher susceptibility to dental diseases, vitamin E assists in treating oral mucositis, and vitamin K is essential for blood clotting in dental surgeries. Deficits in B-complex and vitamin C result in enamel hypomineralization and soft tissue ailments, including aphthous stomatitis and gingival petechiae. Additionally, a lack of vitamin B7 compromises the immune response, increasing oral candidiasis risk. Therefore, vitamin deficiencies markedly affect children’s oral health, highlighting the need for joint efforts between dental professionals and caregivers for effective pediatric care. Addressing vitamin deficiencies through supplementation and tailored dental care emphasizes the significance of nutritional health in children’s overall and dental well-being, advocating for a collaborative approach to achieve optimal health outcomes.

## 1. Introduction

**Figure 1:**
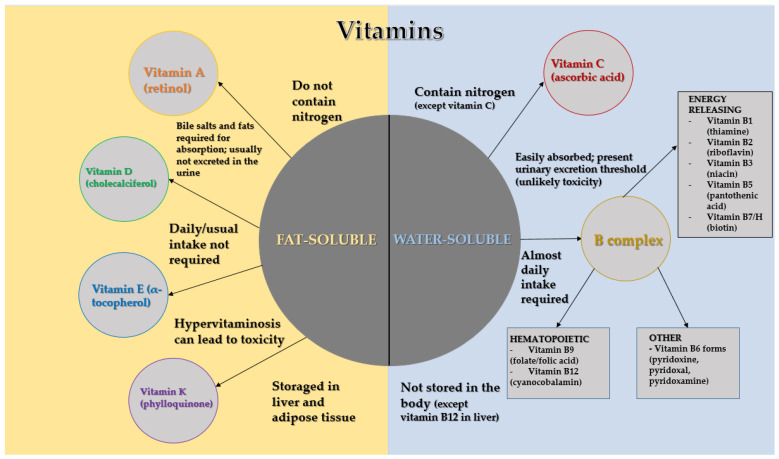
Vitamin classification by solubility and properties (an original scheme reproduced from Hugar et al. [1] and Mataix [2].

Vitamins, diverse in chemical composition and essential for the proper functioning of living beings, are indispensable organic compounds found in a variety of foods. These substances, not synthesized internally, serve as pivotal biocatalysts in human nutrition, facilitating numerous physiological processes and being fundamental for the optimal growth and development of children. They hold significant value in the prophylaxis and treatment of a range of diseases, infections, and certain cancers, as well as in the nutritional enhancement and stabilization of food items, alongside their role as antioxidants [1,2,3]. Vitamins are categorized based on their solubility into two groups: fat-soluble vitamins (A, D, E, K), which are crucial for cell membrane fluidity, and water-soluble vitamins (B-group vitamins and vitamin C), which are essential for enzyme activation [4,5,6,7] (Figure 1). While an overdose of vitamins might lead to hypervitaminosis, a condition associated with various health issues, a lack of vitamins, or avitaminosis, poses a more frequent threat [1,2,3,4,5,6,7]. Insufficient vitamin intake can impair several bodily functions, resulting in conditions such as night blindness, clotting problems, and bone diseases like rickets and osteomalacia, as well as deterioration of nerve and muscle health [3,8]. These insufficiencies also compromise the dental health of children, evident through changes in both hard and soft oral tissues, manifesting as enamel defects, cheilitis, glossitis, and gingivitis [9,10]. Often, these oral signs in children stem from extended periods of vitamin scarcity. The role of vitamins is paramount across all life stages, particularly in childhood nutrition, to foster the establishment of sound dietary habits influenced by familial or community nutritional practices [3,11]. The Croatian Pediatric Society, as well as international health organizations, including the World Health Organization (WHO) and the Centers for Disease Control and Prevention (CDC), underscore the criticality of averting vitamin and mineral shortages from infancy to early childhood, advocating for enhanced vitamin consumption during these crucial phases [12,13,14]. Contemporary lifestyles, coupled with dietary preferences such as veganism and vegetarianism, frequently result in vitamin inadequacies. Highlighting straightforward strategies for a nutritious and balanced diet is essential to circumvent these deficiencies [3,8,11].

### Purpose of the Study

The purpose of this manuscript is to explore the current understanding of the oral manifestations of vitamin deficiencies in children.

## 2. Materials and Methods

In conducting this review, we employed a systematic approach to data collection to ensure a comprehensive examination of the current literature on the oral manifestations of vitamin deficiencies in children. The literature search was methodically performed using the PubMed database, targeting publications between the years 2000 and 2024. This timeframe was chosen to focus on the most recent and relevant studies, reflecting contemporary research findings and professional guidelines. Inclusion criteria were: (1) studies that explicitly explore the relationship between vitamin deficiencies and oral health issues in children, including research on oral symptoms (such as enamel defects, cheilitis, glossitis, and gingivitis) attributable to vitamin insufficiency; (2) articles providing statistical analyses of the prevalence, risk factors, and outcomes of vitamin deficiencies affecting pediatric oral health; (3) professional advisories and guidelines from recognized bodies that offer insights into best practices for diagnosing and managing vitamin-related oral health issues in children; and (4) research focusing on pediatric cases and hypoplastic teeth associated with vitamin deficiencies. Exclusion criteria were: (1) publications dated before the year 2000, to ensure the relevance and recency of the data included in our review; (2) studies not specifically addressing pediatric populations or not focusing on oral health outcomes related to vitamin deficiencies; (3) articles lacking empirical evidence or statistical analysis supporting their findings; (4) non-peer-reviewed literature, to maintain the scientific rigor and reliability of the information reviewed.

Our selection process was geared towards peer-reviewed articles, including original research papers, systematic reviews, meta-analyses, and professional guidelines. The aim was to encompass a wide range of high-quality evidence, ensuring a thorough understanding of the topic at hand.

## 3. Fat-Soluble Vitamins

Vitamins A, D, E, and K form a group of fat-soluble vitamins, each endowed with distinct attributes crucial for maintaining an individual’s health. These vitamins participate in intricate processes of assimilation, metabolic transformation, and systemic distribution, safeguarding cellular integrity and bolstering organ function. Their mechanisms of action include improving cell membrane fluidity and facilitating transport, as well as engaging in oxidation–reduction (redox) reactions. Upon consumption and metabolic processing, these vitamins are retained within the body’s reserves for future utilization. Their primary sources include a diverse array of fruits, vegetables, nuts, and animal products, distinguished by their ability to dissolve in fats and oils rather than water, a property known as lipophilicity [1,2,4,5]. The requisite daily intake of these vitamins is contingent upon a child’s age, highlighting the significance of adhering to dietary recommendations to prevent deficiencies. Vitamin dosage is quantified in International Units (IU), reflecting the amount of a substance with biological efficacy. Higher dosages are denoted in micrograms (μg) or milligrams (mg), underscoring the necessity of following dietary guidelines to safeguard the health and development of children [5,15] (Table 1).

### 3.1. Vitamin A

Vitamin A, comprising retinoids, is available in two primary forms: preformed vitamin A (including retinol, retinaldehyde, and retinoic acid) and provitamin A (carotenoids) [17]. This vitamin group, featuring retinol and beta-carotene, is naturally present in animal sources like fish liver oil, milk, dairy, egg yolk, and a range of yellow and green fruits and vegetables [18]. Retinol is imperative for rhodopsin production, aiding vision in low light, and is essential for immune function, skin and bone health, and growth [19]. Retinoic acid, another form of vitamin A, plays a crucial role in immune defense and tolerance in the intestines via its interaction with nuclear receptor RAR and kinase signaling pathways, and it is vital for every phase of wound healing, promoting tissue and fibroblast growth, angiogenesis, collagen synthesis, and more [20,21]. Both topical and systemic vitamin A supplementation has been shown to augment collagen deposition in the skin [19]. It also supports the development of the ectoderm in fetuses and is crucial for fetal growth [15].

#### Oral Manifestations of Vitamin A Deficiency

**Figure 2:**
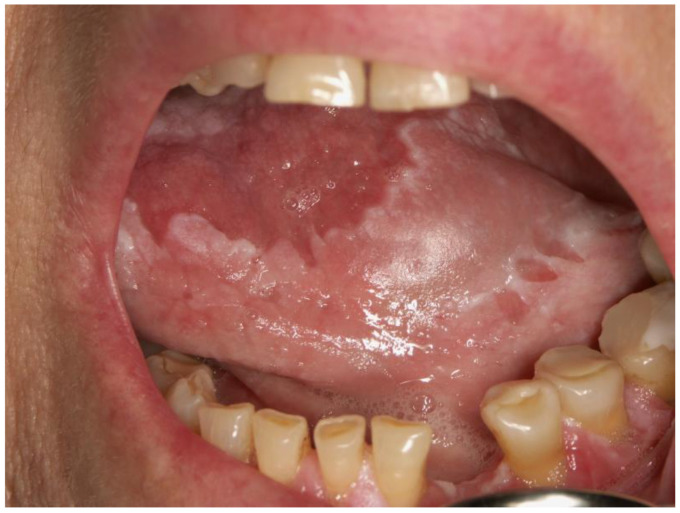
A white keratotic lesion extended along the sublingual mucosa and the lateral edge and ventral part of the tongue, which can also be seen as individual foci on the gingiva. The peculiarity of these white lesions is that they cannot be scraped off. Hypovitaminosis A is a common cause of oral keratotic changes and disorders of mucosal keratinization (Courtesy of Prof. Dr. Marinka Mravak-Stipetić).

Vitamin A deficiency (VAD) is predominantly observed in infants and preschool-aged children, attributed to low retinol stores at birth and increased nutritional needs during rapid growth periods. VAD is linked to significant child and maternal mortality in developing regions, affecting approximately 1–2.5 million individuals annually [22,23]. Children suffering from VAD may exhibit symptoms like night blindness, keratomalacia, xerophthalmia, Bitot spots, and follicular hyperkeratosis, along with increased susceptibility to infections and nail fragility [18]. Oral complications from VAD include oral keratotic changes and disorders of mucosal keratinization, as well as enamel and dentin anomalies, leading to an increased risk of dental caries, enamel hypoplasia, and periodontitis (Figure 2).

Enamel damage, or amelogenesis imperfecta, can result in hypoplasia or opacity, raising the risk for early childhood caries due to compromised enamel integrity. Additionally, dentin defects may show abnormal calcifications and alterations in the dental pulp’s structure [21,24,25,26]. Less frequently, VAD can lead to fungal oral infections, characterized by white, soft plaques on the tongue and oral mucosa, complicating feeding and swallowing [27]. While VAD is a significant concern in regions with rice-based diets, like Southeast Asia and Africa, Croatia does not require routine vitamin A supplementation for healthy children, except for those with fat absorption difficulties. A diet rich in fruits and vegetables can ensure adequate vitamin A intake, with vegan diets providing sufficient levels through carotenoids in orange and yellow produce [28].

### 3.2. Vitamin D

Vitamin D, a steroid hormone, includes two primary forms beneficial for humans: cholecalciferol (vitamin D3) and ergocalciferol (vitamin D2), synthesized in the skin under sunlight exposure [29]. This vitamin is essential for regulating the balance of calcium and phosphorus in the bloodstream, facilitating their absorption in the intestines and reabsorption in the kidneys [30,31]. Its widespread presence of receptors across various cell types has spurred research into its potential links with numerous diseases [32]. Vitamin D is particularly known for promoting immune tolerance through its action on dendritic cells, encouraging the development of regulatory T cells [20]. It plays a pivotal role in maintaining the health of the neuromuscular, skeletal, dermal, and cardiovascular systems [33]. Furthermore, vitamin D is recognized for its tumor-suppressing, anti-inflammatory, and antibacterial capabilities, along with its contribution to calcium absorption and bone remodeling [34,35]. Children primarily obtain vitamin D from sources like fish oil, egg yolk, vitamin-fortified margarine, and vitamin-enriched baby foods [15,18,36].

#### Oral Manifestations of Vitamin D Deficiency

**Figure 3:**
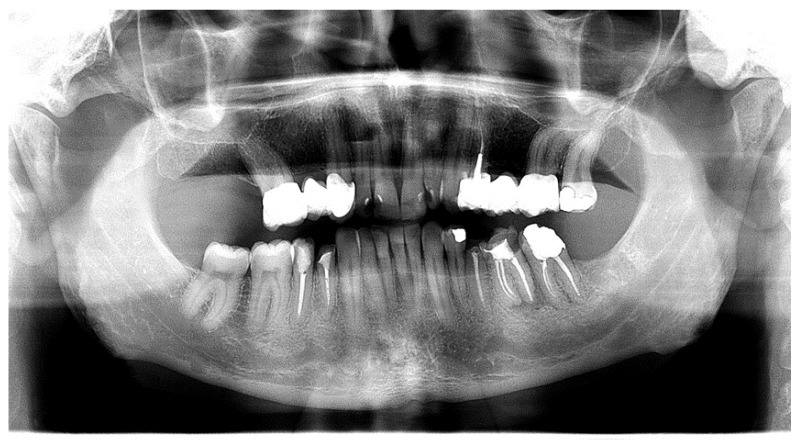
Image shows a decrease in bone mineral density in the mandible, particularly along the mandibular canal and an increase in alveolar porosity due to Vitamin D deficiency (Courtesy of Prof. Dr. Marinka Mravak-Stipetić).

Awareness around Vitamin D deficiency (VDD) has grown significantly due to its widespread occurrence [37], raising concerns particularly in pediatric health, as highlighted by Aguiar et al. [38]. VDD is notably severe in infants, stemming from low levels in both maternal and cow’s milk. In older children, deficiencies often result from inadequate dietary choices, with chronic shortages seen in those following vegan diets. The primary consequence of VDD is impaired bone mineralization, manifesting as rickets in young children or osteomalacia in adolescents. Oral impacts of this deficiency include a form of amelogenesis imperfecta during tooth development, alterations in dentin leading to dentogenesis imperfecta, and ectodermal dysplasia. VDD can also trigger decreased bone mineral density, leading to jawbone resorption [15,21] (Figure 3).

Amelogenesis imperfecta, affecting enamel, can be linked to deficiencies in vitamins A and D or genetic factors, presenting as discoloration and structural alterations. Enamel hypoplasia, characterized by white or yellow-brown spots, primarily affects primary teeth and can lead to defects in permanent successors. This condition not only alters appearance but also increases sensitivity and caries risk [39]. Ectodermal dysplasia in the oral cavity is identified by the presence of fewer teeth of irregular shapes (cone-shaped), resulting in smaller teeth and potentially leading to asymmetrical alveolar ridge development. These cone-shaped teeth are often hypoplastic [40]. Vitamin D supplementation is globally recognized for its role in preventing rickets during infancy. The developmental stages of fetal teeth are vitamin D-dependent, suggesting the maternal vitamin D status during pregnancy influences tooth mineralization [41]. While earlier studies found no connection between vitamin D and cariogenic activity [42,43], recent research suggests that vitamin D supplementation can prevent the onset and progression of dental caries, recommending its use in children at risk of severe early-childhood caries [44,45]. High-dose vitamin D supplementation during pregnancy has been associated with a reduced risk of enamel defects in newborns [45], underscoring its preventative role against enamel deficiency. Vitamin D also plays a crucial role in the immune system, with optimal levels linked to lower chances of dental caries [46,47]. However, research findings have been mixed regarding this association [48,49]. Adequate vitamin D intake can mitigate gingivitis risks or reduce bacteria in gingival inflammation, thanks to its immunosuppressive and anti-inflammatory properties, which are important for preventing infections in the oral cavity and initiating cell apoptosis. Vitamin D influences the immune system by inducing human cathelicidin (LL-37) in oral epithelial cells, which has antimicrobial and antiendotoxin activities. Children with high caries activity show low concentrations of LL-37, emphasizing its role as a “guardian of the oral cavity” and its importance in oral health [50,51,52,53,54]. Maternal vitamin D deficiency is linked to increased DMFT scores in children aged 12–35 months [55]. Vitamin D’s effects on bone metabolism and its potential anti-inflammatory properties make it beneficial in treating periodontitis and enhancing postoperative wound healing following periodontal surgery. It acts as a potent signaling agent for alveolar bone resorption and is associated with lower levels of pro-inflammatory markers [56,57,58]. Given the myriad issues stemming from VDD in children, it has emerged as a public health priority, especially in developed nations. The current lifestyle and rising obesity rates among children exacerbate the risk of vitamin D deficiency. Prophylactic vitamin D3 doses range from 400 IU/day to 1000 IU/day for infants, as per European and American guidelines, emphasizing the need for supplementation throughout the first year of life [30]. Later, dietary sources rich in vitamin D should be encouraged, although consensus on adolescent vitamin D requirements remains elusive [32]. Special attention is warranted for children at higher risk of deficiency, including those with dark skin, obesity, limited outdoor activity, and certain health conditions, necessitating periodic vitamin D level monitoring [28,59].

### 3.3. Vitamin E

Vitamin E, encompassing tocopherols and tocotrienols, is classified into four chemical variants: alpha, beta, gamma, and delta. Alpha-tocopherol stands out as the most crucial for human health, being the primary form metabolized by the liver, whereas other tocopherols and tocotrienols are largely excreted [60,61,62]. Its predominant role is serving as an antioxidant, vital in conditions of heightened metabolic demand, and it also exhibits anti-inflammatory properties while stimulating naïve T cells [20]. Tocopherols are primarily found in vegetable oils from cereal germs, green leafy vegetables, avocados, legumes, and nuts [15,36,63].

#### Oral Manifestations of Vitamin E Deficiency

**Figure 4:**
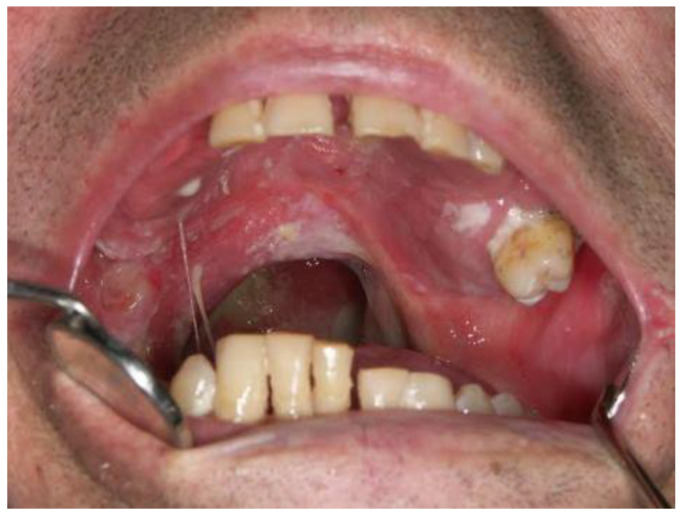
Mucositis and xerostomia in a patient undergoing radiotherapy after surgery of oropharyngeal cancer. Ulcerated and inflamed areas of the oral mucosa can be seen along with thick mucous saliva and white plaque deposits on the mucosa due to lack of serous saliva and washing of mucosa (Courtesy of Prof. Dr. Marinka Mravak-Stipetić).

Owing to its broad distribution in numerous widely consumed foods, vitamin E deficiency is uncommon in the general and developed world populations, with only milder forms observed in South Asian regions [63,64,65]. The groups most at risk include preterm infants, children, and pregnant women, largely due to inadequate fat absorption or metabolic issues [63,65]. During pregnancy, only minimal amounts of vitamin E are transferred through the placenta, resulting in newborns with low vitamin E stores. A severe shortage can lead to neurodegenerative disorders such as ataxia and myopathy, affecting peripheral and motor nerves and the skeletal system [66]. Additionally, it may compromise immune function and trigger hemolytic anemia [64]. In the context of oral health, vitamin E has demonstrated effectiveness in managing oral mucositis, a condition particularly prevalent among children and adults undergoing cancer chemoradiotherapy (Figure 4). This condition involves painful ulcerations in the mouth that can significantly hinder feeding. Vitamin E, used either singly or alongside vitamin A, has been employed as a therapeutic agent [26,67]. While its impact on periodontal health may not be as pronounced as other vitamins, vitamin E is acknowledged for its capacity to modulate inflammation within the oral cavity, offering a beneficial effect in managing oral health conditions [63,65].

### 3.4. Vitamin K

Vitamin K is indispensable for numerous blood coagulation pathways, with K1 (phylloquinone) and K2 (menaquinone) being crucial for human health [67,68]. Vitamin K1 is produced by intestinal bacteria, while a well-rounded diet ensures a sufficient intake of vitamin K2 [69]. However, the use of antibiotics can disrupt the balance of this essential nutrient [70]. Foods rich in vitamin K1 include liver and green vegetables, while vitamin K2 is found in dairy products [15,18,65].

#### Oral Manifestations of Vitamin K Deficiency

**Figure 5:**
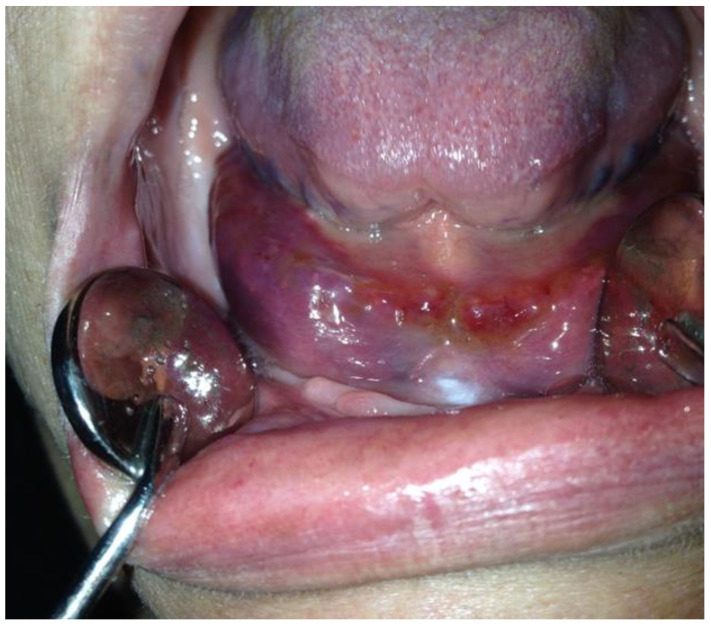
Submucosal bleeding manifesting as a sublingual hematoma in patients with a blood coagulation disorder due to vitamin K deficiency (Courtesy of Prof. Dr. Marinka Mravak-Stipetić).

At birth, newborns exhibit low levels of vitamin K due to the limited ability of this vitamin to cross the placenta. Vitamin K is vital for the synthesis of blood clotting factors, and its deficiency may result in neonatal hemorrhagic disease. This condition is characterized by sudden and potentially life-threatening bleeding in various parts of the body, including the brain, skin, and digestive tract, which could have severe or even fatal neurological consequences [71]. To mitigate this risk, vitamin K supplementation is administered immediately after birth and continued through the third month of life, especially for exclusively breastfed infants, due to the low levels of vitamin K in breast milk [28]. Surveillance efforts and educational campaigns are essential to prevent vitamin K deficiency bleeding (VKDB), a largely preventable condition that has resulted in fatalities, particularly in cases of home births and parental refusal of vitamin K prophylaxis [71]. Zellweger spectrum disorders (ZSDs), which involve a disruption in peroxisome biogenesis, leading to various metabolic issues, also highlight the potential complications of vitamin K deficiency, such as hepatic dysfunction and coagulopathy, often presenting as bleeding complications in affected patients (Figure 5).

These issues can be partially alleviated through oral or IV vitamin K supplementation, which improves overall vitamin K status and blood clotting capabilities [72,73]. Individuals with cystic fibrosis or those undergoing anticoagulant therapy are at increased risk for vitamin K deficiency, affecting coagulation and elevating the risk of bleeding during dental procedures. This underscores the importance of careful management and supplementation in these populations [74,75]. Furthermore, dietary supplements combining vitamins K1 and D3 are marketed for their synergistic effects on bone health and the development of a robust skeletal system in children, underscoring the broad utility and significance of vitamin K in human nutrition and health [28].

## 4. Water-Soluble Vitamins

Water-soluble vitamins encompass the B-complex group (vitamins B1, B2, B3, B5, B6, B7, or vitamin H, B9, and B12) along with vitamin C, all of which play pivotal roles in human health [2,6]. These vitamins are readily absorbed through the intestines and are sourced from a diverse array of foods, including fruits, vegetables, dairy products, legumes, meats, eggs, and cereals. The occurrence of a complete deficiency in all water-soluble vitamins is uncommon but may be seen in specific populations such as alcoholics, individuals with malabsorption syndromes, those adhering to a vegan diet without proper supplementation, and in cases of malnutrition [6,7]. The recommended daily intake of these essential nutrients tends to increase with age and is quantified in micrograms (mcg) and milligrams (mg), underscoring the importance of a balanced diet in maintaining optimal levels of these vitamins (Table 2).

### 4.1. B-Complex Vitamins

B-complex vitamins, comprising B1 (thiamin), B2 (riboflavin), B3 (niacin), B5 (pantothenic acid), B6 (pyridoxine), B7 (biotin or vitamin H), B9 (folate), and B12 (cobalamin), are foundational to cell metabolism and replication and are vital for neurological functions. These vitamins are crucial for cytotoxic cellular immunity, modulating T cell responses, and are found in a wide variety of foods, including yeast, meat, legumes, whole grains, nuts, offal, dairy products, seeds, fish, eggs, green vegetables, and sprouted grains [20,76,77]. They are also frequently added to processed foods such as flours, rice, pasta, and cereals, with vitamin B2 commonly used as a coloring agent for its yellow hue [15,28].

#### Oral Manifestations of B-Complex Vitamin Deficiency

**Figure 6:**
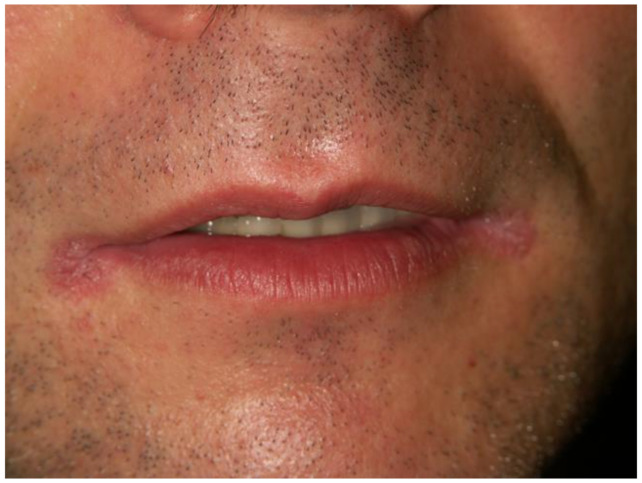
Angular cheilitis due to hypovitaminosis of B2 vitamine (Courtesy of Prof. Dr. Marinka Mravak-Stipetić).

While deficiencies in B-group vitamins are uncommon in children due to their broad availability in the diet, deficiencies can impact the immune, cardiovascular, and nervous systems. Thiamin (vitamin B1) is essential for energy production, nerve impulse transmission, and the maintenance of the myelin sheath. Severe deficiency can lead to diseases such as beriberi and Wernicke–Korsakoff syndrome, especially in individuals with high rice consumption, chronic alcoholism, or those suffering from malnutrition and malabsorption syndromes [65,78,79]. Riboflavin (vitamin B2) is key for energy metabolism and is found in high concentrations in yeast extract, organ meats, wheat bran, milk products, eggs, and meat. It is notably sensitive to light, with its degradation accelerated by the presence of sodium bicarbonate during cooking [36]. Oral health can be significantly affected by deficiencies in vitamins B1 and B2, leading to conditions such as recurrent aphthous stomatitis (RAS), glossitis, and angular cheilitis (Figure 6).

These deficiencies may also impair postnatal amelogenesis, resulting in enamel hypomineralization [76,80,81,82]. RAS presents as painful ulcers on the non-keratinized oral mucosa, affecting speech and eating, with the minor form being most common in children [83,84]. This condition, which may also be related to systemic diseases such as celiac disease, underscores the importance of evaluating children with RAS for nutritional status and considering screening for underlying conditions in cases of hematological abnormalities [85]. Effective management includes topical steroid therapy, good oral hygiene, and supplementation with essential nutrients, including B1, B2, B6, B12, and vitamin C [65,83,84]. Glossitis, characterized by inflammation and changes in the tongue’s appearance and texture, can also result from deficiencies in vitamins B1 and B2. Treatment involves nutritional supplementation to address the underlying deficiency [86]. 

**Figure 7:**
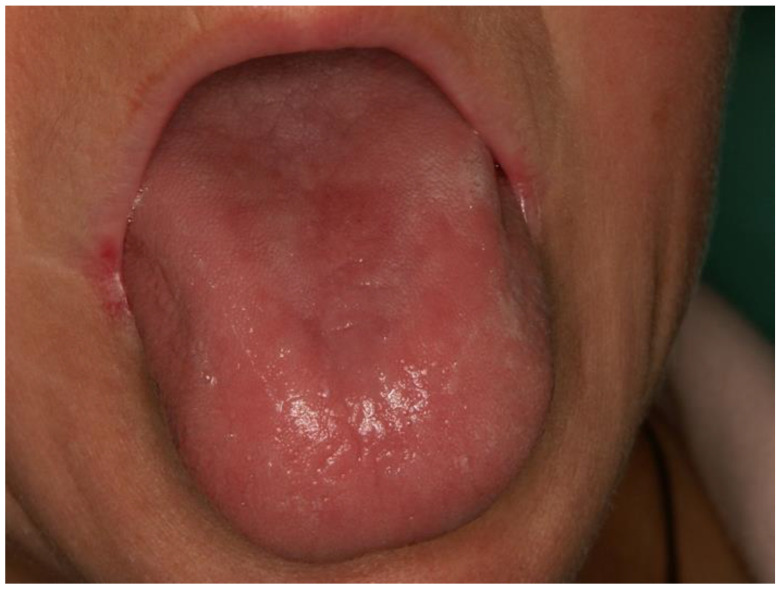
Exfoliative glossitis and angular cheilitis due to hypovitaminosis of B2 and B6 vitamins. Inflammatory changes in the tongue mucosa are usually accompanied by a burning symptom, which is particularly pronounced in vitamin B1 deficiency (Courtesy of Prof. Dr. Marinka Mravak-Stipetić).

Deficiency of vitamin B3, known as niacin, results in pellagra, characterized by dermatological manifestations. Historically prevalent in the early 1900s among populations consuming niacin-deficient corn-based diets, pellagra is identified by dermatitis, glossitis, unpleasant breath odor due to bacterial growth or dry mouth, cheilitis, and RAS [87,88]. Pantothenic acid, or vitamin B5 deficiency, often occurs in those who are highly physically active, such as athletes, or suffer from severe undernutrition. Rich dietary sources of B5 include mushrooms, legumes, eggs, alfalfa, avocados, dairy, organ meats like liver, kidney, and heart, as well as whole grains and yeast [18,36]. Symptoms manifest as headaches, fatigue, muscle cramps, paresthesia, and nausea [89]. B5 plays a significant role in managing dry mucosal conditions, including xerosis and cheilitis, with its deficiency leading to similar oral complications observed with other B vitamins [90]. Vitamin B6, or pyridoxine, is essential for embryonic development and early childhood, contributing to hemoglobin synthesis, amino acid metabolism, and protein synthesis. Although it is added to various multivitamin supplements and food items, making deficiency rare, it can lead to poor absorption in the gastrointestinal tract, liver disorders, weakened immunity, and dermatological issues. Oral symptoms in children due to B6 deficiency encompass angular cheilitis, glossitis, RAS, and halitosis (Figure 7). Immune system decline may also precipitate fungal infections in the oral cavity, akin to those seen with vitamin A shortages [91,92].

**Figure 8:**
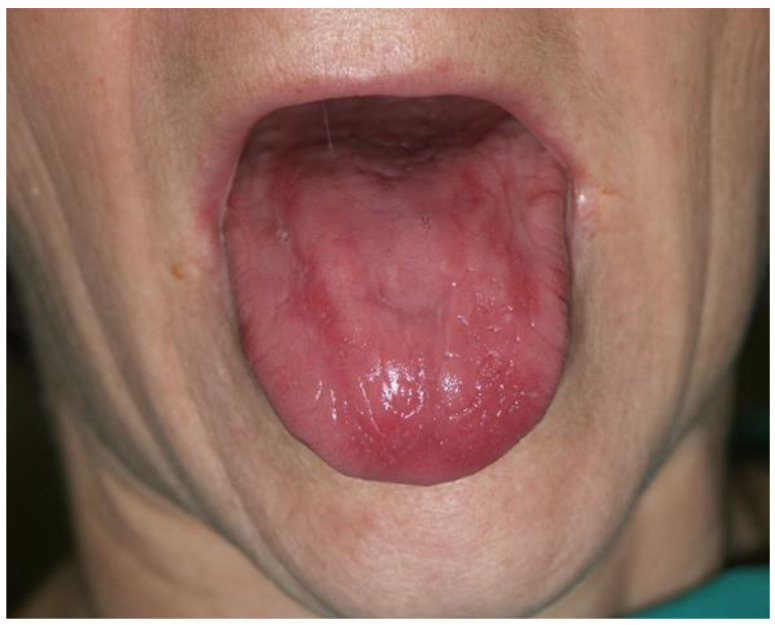
Exfoliative (Moeller Hunter) glossitis. Inflammation and complete loss of filiform papillae of the dorsal tongue mucosa in patients with pernicious anemia due to vitamin B12 deficiency (Courtesy of Prof. Dr. Marinka Mravak-Stipetić).

Biotin (vitamin H or B7) serves as a vital coenzyme for the metabolism of fats, carbohydrates, and amino acids, supporting cellular proliferation and the health of hair and nails [21,93]. Despite its abundance in egg yolk, offal, yeast, mushrooms, bananas, and peanuts, deficiency can occur from consuming raw egg whites, which inhibit biotin absorption, or from prolonged antibiotic use [36,91,94]. Biotin deficiency symptoms include dermatitis, hair loss, anemia, depressive symptoms, vomiting, and nail inflammation [18]. In children, it may lead to conjunctivitis, ataxia, developmental delays, muscle weakness, paralysis, and vision issues [18]. Oral candidiasis, caused by Candida albicans, is a notable symptom, presenting as white patches within the oral cavity that can hinder swallowing [27]. To counteract biotin and other vitamin deficiencies, the market provides mixed vitamin and mineral supplements, particularly beneficial for selective eaters, ensuring up to 100% of daily nutritional needs when consumed in appropriate volumes [95]. Folic acid (vitamin B9) is imperative in the diets of pregnant women for DNA synthesis and fetal development, with deficiencies previously observed in infants consuming goat’s milk. Nowadays, breastfeeding and fortified formulas have significantly reduced this risk. Lack of B9 leads to megaloblastic anemia and is especially critical for children with gastrointestinal disorders such as celiac disease, Crohn’s disease, and ulcerative colitis [96]. Oral symptoms of B9 deficiency include gingivitis, characterized by swollen and bleeding gums, as well as angular cheilitis and glossitis, mainly affecting the soft tissues of the mouth [28,97]. Cyanocobalamin, or vitamin B12, plays a pivotal role in several critical bodily functions, including nerve cell function, DNA replication, and the production of mood-regulating neurotransmitters. It is also integral to managing homocysteine levels, which, when elevated, are associated with an increased risk of cardiovascular diseases. B12 deficiency can lead to pernicious anemia and chronic fatigue, significantly impacting intellectual and neurological development [98]. It acts as a crucial cofactor in the human body for two specific enzymatic reactions: the conversion of homocysteine to methionine in the cytosol and the conversion of methylmalonyl CoA to succinyl-CoA in the mitochondria. Interruptions in these processes can trigger a B12 deficiency [98]. The effects and severity of a B12 deficiency vary, influenced by the deficiency’s extent and duration. It predominantly affects the blood, bone marrow, and nervous system, leading to megaloblastic anemia due to impaired DNA synthesis in rapidly dividing cells. Neurological manifestations can range from issues with myelin synthesis and repair to cognitive decline and psychosis [98]. Notably, vitamin B12 deficiency is reported in 10–50% of women of childbearing age and pregnant women globally [99]. Newborns might not show symptoms at birth but can develop significant, potentially irreversible, multisystemic issues, including developmental delays, later in infancy. Early diagnosis and treatment in such cases can facilitate normal development and also benefit mothers who were previously undiagnosed [100]. For primary prevention of B12 deficiency, systematic supplementation during pregnancy is advised, especially for those without gastrointestinal malabsorption issues. Early pregnancy assessments for B12 levels can aid in identifying and treating asymptomatic mothers with atrophic gastritis, a condition marked by severe B12 deficiency [99]. Vitamin B12 is primarily found in animal products like milk, eggs, and fish, and is less common in plant-based sources, placing vegetarians, vegans, and their infants at a heightened risk for deficiency. Fortification of cereals, yeast, and some beverages with B12 has been implemented to address this gap [91]. The Croatian Pediatric Society advocates for B12 supplementation among breastfeeding vegan mothers [12], emphasizing its necessity for preventing anemia and its oral manifestations such as mucosal pallor, anemic gingiva, glossitis, dry mouth, and taste alterations in vegan children [28,35,93,101] (Figure 8).

Research by Khan et al. [102] and Liu et al. [103] highlighted the prevalence of haematinic deficiencies in patients with RAS, with significant reductions observed in serum folic acid and B12 levels. Consequently, inflammation of the lips and perioral skin can often be linked to nutritional deficiencies, including B12 and sideropenic anemia, which are implicated in the etiology of exfoliative cheilitis [104,105]. These findings underscore the importance of evaluating vitamin and mineral status in patients presenting with inflammation of the lips, perioral skin, or oral cavity, particularly in persistent cases.

### 4.2. Vitamin C

Vitamin C, also known as ascorbic acid, is integral to the metabolism of carbohydrates, proteins, and lipids, and plays a pivotal role in the body’s redox reactions [106]. It serves as a cofactor in numerous enzymatic processes essential for the growth, repair, and development of tissues [107], including the hydroxylation of collagen, a critical step in connective tissue repair [108]. Moreover, vitamin C is involved in the biosynthesis of carnitine and norepinephrine, the metabolism of tyrosine, and the amidation of peptide hormones [109]. Its primary role involves the maintenance of collagen, vital for the regeneration of skin, bones, and ligaments, and it significantly contributes to wound healing, serves as an antioxidant, and plays a crucial role in preventing bacterial infections [110]. Beyond its antioxidant capabilities, vitamin C influences cell signaling and epigenetic modifications [20]. Rich sources of vitamin C include citrus fruits, green leafy vegetables, and bell peppers [93,111].

#### Oral Manifestations of Vitamin C Deficiency

**Figure 9:**
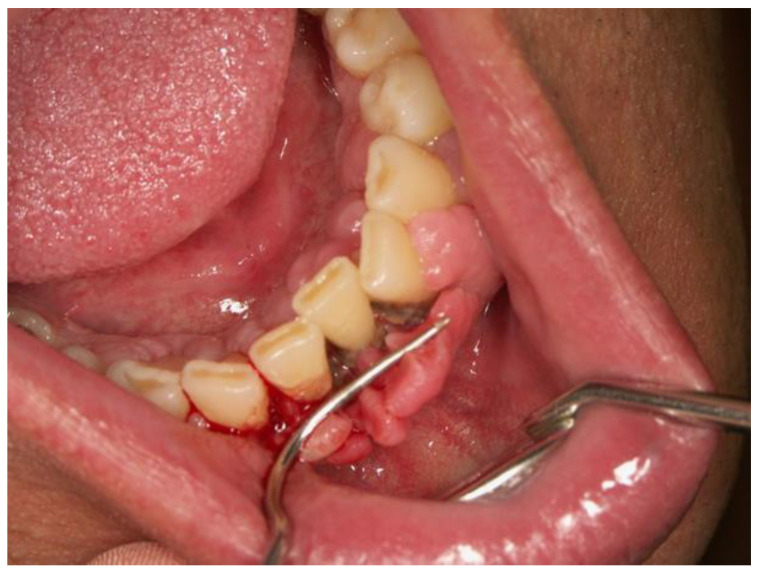
Hyperplastic gingivitis and periodontitis. Inflammation and hypertrophy of gums with bleeding and deep periodontal pockets as a result of hypovitaminosis C (Courtesy of Prof. Dr. Marinka Mravak-Stipetić).

In children, vitamin C deficiency can arise from restrictive eating patterns, including anorexia and bulimia. It is essential for iron absorption, a vital mineral for hematopoiesis. Vitamin C is thermolabile, meaning its content diminishes with cooking [111]. Scurvy, the disease caused by vitamin C deficiency, though often considered a historical condition, persists today, especially among children with neurodevelopmental issues or selective diets. The diagnosis of scurvy can be challenging due to its rarity and the diversity of its nonspecific symptoms, which include gingival changes [112]. Vitamin C is also crucial for immune function, with a noted increase in infection susceptibility in deficient individuals; it is even considered for adjunctive sepsis therapy [113]. Signs of deficiency typically emerge after 30 to 90 days of inadequate intake, with clinical symptoms reflecting its various metabolic roles [114,115]. These symptoms can mimic rheumatological, infectious, or hematological conditions due to their musculoskeletal and mucocutaneous manifestations [116]. Scurvy is characterized by gum hypertrophy, swelling, bleeding, follicular hyperkeratosis, extremity swelling, poor wound healing, and petechiae [117]. Oral signs are particularly indicative of scurvy, though gingival overgrowth can also suggest other conditions, necessitating careful differential diagnosis [118,119] (Figure 9). As mentioned, in scurvy, gums become swollen, tender, and bleed easily, which leads to tooth loss and secondary infections as wounds heal poorly [120]. Despite being rare in developed countries, scurvy occurs in individuals at risk, such as the elderly, those with malabsorption syndromes, eating disorders, and children with restrictive diets [121].

As humans cannot synthesize vitamin C, it must be obtained through diet [122]. Serum vitamin C levels, a standard diagnostic test, reflect recent dietary intake and can indicate the risk of developing scurvy [123]. The resurgence of scurvy in children, especially those with neurodevelopmental disorders and selective diets, highlights the need for dietary guidance and supplementation [124]. Post-surgical applications of vitamin C-enriched pastes/gels have been shown to enhance wound healing, and supplementation with vitamins A, B, E, and omega-3 fatty acids improves outcomes following periodontal interventions [65]. Vitamin C supplementation can alleviate gingival inflammation by enhancing collagen stabilization and reducing bleeding and inflammation [65]. Deficiency may also predispose children to fungal infections of the oral mucosa and xerostomia due to decreased salivary secretion [28,93,106]. Furthermore, vitamin C is beneficial in managing oxidative stress associated with periodontal diseases, a response to bacterial toxins causing mucosal damage in gingivitis and periodontitis [65,107,125].

## 5. The Importance of Recognizing Vitamin Deficiencies in the Oral Cavity

Vitamins, as indispensable micronutrients, play a vital role in supporting the myriad of biological processes within the human body and are essential for the healthy growth and development of children. Nutritional advice has become a cornerstone of preventive dentistry, significantly impacting long-term oral health outcomes [21,126]. Early detection of vitamin deficiencies during childhood is a key focus in pediatrics and pediatric dentistry to ward off oral, dental, and craniofacial diseases. An integral aspect of understanding the relationship between vitamin deficiencies and oral health, particularly in the development of dental anomalies, involves the consideration of vitamin absorption processes and potential concomitant systemic pathologies. For instance, dental fluorosis, characterized by changes in the appearance of tooth enamel in children exposed to high levels of fluoride during teeth development, has been the subject of increased scrutiny [127]. Recent studies have begun to explore how certain vitamins might influence the body’s response to fluoride and the subsequent development of fluorosis. For instance, research indicates that adequate levels of vitamin D might reduce the risk of dental fluorosis by enhancing calcium metabolism, thus promoting healthy tooth mineralization and potentially counteracting fluoride’s adverse effects [128]. Furthermore, the antioxidant properties of certain vitamins might offer protective mechanisms against oxidative stress induced by fluoride, suggesting a complex interplay that warrants further investigation [129]. These findings underscore the importance of a balanced nutritional status in children, not only to prevent vitamin deficiencies but also as a potential factor influencing the risk and severity of dental fluorosis. Moreover, the efficiency of vitamin absorption can significantly influence the severity and manifestation of vitamin-related oral health issues, including dental anomalies. This is because systemic conditions often affect the body’s ability to absorb, metabolize, and utilize essential vitamins, thereby exacerbating the risk of deficiencies. Several systemic pathologies, such as gastrointestinal disorders including celiac disease, Crohn’s disease, and cystic fibrosis, have been identified as critical factors that impair vitamin absorption. For instance, celiac disease affects the small intestine’s ability to absorb nutrients from food, leading to a deficiency in various vitamins [130]. Similarly, Crohn’s disease and cystic fibrosis can result in malabsorption syndromes, directly impacting the body’s vitamin levels [131,132]. The presence of such systemic conditions is therefore crucial in assessing the risk and development of dental anomalies linked to vitamin deficiencies. Vitamins A, D, E, and K, which are fat-soluble, as well as the B-group vitamins and vitamin C, play pivotal roles in oral health. For example, vitamin D’s role in calcium and phosphate metabolism is essential for dental development and bone health, with deficiencies linked to enamel defects and an increased risk of dental caries [44]. The impact of systemic diseases on these vitamins’ absorption underscores the complexity of diagnosing and managing dental anomalies associated with vitamin deficiencies. Given the intricate relationship between systemic health, vitamin absorption, and oral health, a holistic approach to patient assessment is essential. This should include a thorough examination of dietary habits, gastrointestinal health, and a comprehensive medical history to identify any underlying conditions that may affect vitamin status. Incorporating such considerations into clinical practice and research can enhance our understanding of the etiological factors contributing to dental anomalies and guide more effective prevention and treatment strategies. Adopting a nutritious diet is a pivotal strategy in enhancing oral and systemic health, where altering dietary and lifestyle habits can foster the amelioration of these conditions [133]. Modifying diet represents a readily adjustable factor, crucial not just for oral health but also for systemic well-being, including its influence on the autologous self-renewal of stem cell niches related to oral mucosal integrity [134,135]. However, genetic variations among populations may significantly affect vitamin assimilation [136], emphasizing the concept of biochemical individuality and the toxicological principle that the dose determines the poison. Assessing a vitamin’s therapeutic efficacy involves considering the dosage, form, source, bioavailability, and interactions with other nutrients. Child malnutrition, including hypovitaminosis, can stem from adverse cultural practices, environmental degradation, gender inequality, healthcare accessibility, lack of education, family size, overpopulation, and poverty [137]. Upon noticing initial signs of vitamin deficiency in the oral cavity, immediate actions should be taken to rectify the deficiency and address its dental consequences. This includes supplementing the deficient vitamin and potentially initiating dental interventions such as endodontic or restorative treatments, especially critical for conditions exacerbated by chronic vitamin D deficiency, affecting caries incidence and periodontal disease progression [45,58]. Childhood represents a critical period for instilling proper dietary habits, influenced by familial and societal practices. Modern lifestyles often contribute to inadequate vitamin consumption. The role of parents is crucial in setting dietary examples, promoting family meals, discouraging meal skipping, and offering diverse, healthy food options without resorting to food as a reward or bribe. However, the challenge remains in educating children on balanced nutrition amidst the prevailing fast-food culture, the marketing of unhealthy foods, and dietary habits centered on convenience. Particularly, special diets like veganism and vegetarianism can lead to nutritional deficiencies, as evidenced by numerous studies. The Croatian Pediatric Society, WHO and CDC emphasize the importance of preventive measures against such deficiencies [12,13,14]. Despite challenges, including the influence of technology on dietary choices, initiatives aimed at integrating healthier food options into educational settings and employing nutritionists to devise child-friendly menus are steps towards improving children’s overall and oral health. Educating both children and parents about the implications of vitamin deficiencies on health is paramount, alongside regular dental check-ups for early identification of vitamin-related oral changes. Dental professionals and healthcare providers must be adept at recognizing signs of vitamin deficiencies in the oral cavity and initiating appropriate interventions to mitigate the effects of such deficiencies.

## 6. Conclusions

Vitamins are indispensable for sustaining life, playing a critical role in maintaining the health and functionality of the body, including the stomatognathic system in children. Manifestations of vitamin deficiencies are observable in both the hard and soft tissues of the oral cavity, underscoring the importance of prevention and early detection in their management. The fields of pediatric dentistry and pediatrics are vital in identifying and addressing these deficiencies early on. Deficiencies in vitamins such as A, D, and K prominently affect the hard tissues within the oral cavity, such as teeth and the jawbone. Conversely, deficiencies in water-soluble vitamins, notably the B-complex and vitamin C, tend to present symptoms primarily in the soft tissues, including the tongue, gingiva, buccal mucosa, and lips. While some deficiencies lead to discernible anomalies in teeth or the alveolar ridge, others might not exhibit specific signs yet play a significant role in treating various conditions and complications within pediatric and general dentistry. The initial approach to managing vitamin deficiencies involves their supplementation, subsequently addressing the resultant changes within the pediatric dental care spectrum. This strategy emphasizes the integral role of nutritional health in the overall well-being and dental health of children, highlighting the collaborative effort required between dental professionals and caregivers to ensure optimal health outcomes.

## Figures and Tables

**Table 1:** Suggested daily intake of fat-soluble vitamins during developmental stages (an original scheme reproduced from Office of Dietary Supplements—National Institutes of Health [16]).

Vitamin	Life Stage						
	Birth–6 Months	7–12 Months	1–3 Years	4–8 Years	9–13 Years	Teen Males 14–18 Years	Teen Females 14–18 Years
Vitamin A (mcg RAE)	400	500	300	400	600	900	700
Vitamin D (mcg/IU)	10/400	10/400	15/600	15/600	15/600	15/600	15/600
Vitamin E (mg)	4	5	6	7	11	15	15
Vitamin K (mcg)	2	2.5	30	55	60	75	75

mcg = micrograms; RAE = retinol activity equivalents; IU = international units; mg = milligrams.

**Table 2:** Suggested daily intake of water-soluble vitamins during developmental stages (an original scheme reproduced from Office of Dietary Supplements—National Institutes of Health [16]).

Vitamin	Life Stage						
	Birth–6 Months	7–12 Months	1–3 Years	4–8 Years	9–13 Years	Teen Males 14–18 Years	Teen Females 14–18 Years
Vitamin B1 (mg)	0.2	0.3	0.5	0.6	0.9	1.2	1
Vitamin B2 (mg)	0.3	0.4	0.5	0.6	0.9	1.3	1
Vitamin B3 (mg NE)	2 **	4	6	8	12	16	14
Vitamin B5 (mg)	1.7	1.8	2	3	4	5	5
Vitamin B6 (mg)	0.1	0.3	0.5	0.6	1	1.3	1.2
Vitamin B7/H (mcg)	5	6	8	12	20	25	25
Vitamin B9 (mcg DFE)	65	80	150	200	300	400	400
Vitamin B12 (mcg)	0.4	0.5	0.9	1.2	1.8	2.4	2.4
Vitamin C (mg)	40	50	15	25	45	75	65

mg = milligrams; NE = niacin equivalents; mcg = micrograms; DFE = dietary folate equivalents. ** only mg without NE.

## Data Availability

Not applicable.
